# Analysis of Periprocedural X-ray Exposure in Transarterial Radioembolization with Glass or Resin Microspheres

**DOI:** 10.3390/diagnostics13243609

**Published:** 2023-12-06

**Authors:** Constantin Ehrengut, Johanna Vogt, Jakob Leonhardi, Emma Carabenciov, Felix Teske, Florian van Boemmel, Thomas Berg, Daniel Seehofer, Thomas Lincke, Osama Sabri, Holger Gößmann, Timm Denecke, Sebastian Ebel

**Affiliations:** 1 Department of Diagnostic and Interventional Radiology, University of Leipzig Medical Center, 04103 Leipzig, Germany; johanna_vogt@outlook.de (J.V.); sebastian.ebel@medizin.uni-leipzig.de (S.E.); 2 Division of Hepatology, Department of Medicine II, University of Leipzig Medical Center, 04103 Leipzig, Germany; 3 Department of Visceral, Transplantation, Thoracic and Vascular Surgery, University of Leipzig Medical Center, 04103 Leipzig, Germany; 4 Department of Nuclear Medicine, University of Leipzig Medical Center, 04103 Leipzig, Germany

**Keywords:** Transarterial Radioembolization, radiation protection

## Abstract

**Background**: Transarterial Radioembolization (TARE) is an effective treatment option for both primary and secondary liver malignancies. However, challenging anatomical conditions can lead to prolonged fluoroscopy times (FT), elevated doses of periprocedural X-radiation (DAP), and increased use of contrast agents (CAs). In this study, we examined the influence of our radiologists’ experience and the choice of microspheres on X-ray exposure and CA doses in TARE. **Material and Methods**: Datasets comprising 161 TARE and 164 preprocedural evaluation angiographies (TARE-EVA) were analyzed. Our study focused on assessing DAP, FT, and CA concerning both microsphere types, the radiologist’s experience, and whether the same radiologist performed both the TARE-EVA and the actual TARE. **Results:** In TARE, the use of resin microspheres resulted in significantly higher FT and CA compared to glass microspheres (14.3 ± 1.6 min vs. 10.6 ± 1.1 min and 43 ± 2.2 mL vs. 33.6 ± 2.1 mL, *p* < 0.05), with no notable differences in DAP (*p* = 0.13). Experienced radiologists demonstrated reduced FT/DAP, with a 19% decrease in DAP and 53% in FT during the evaluation angiography (*p* < 0.05) and a 49% reduction in DAP during the actual TARE (*p* < 0.05), with no statistical differences in FT. Performing TARE and TARE-EVA under the same radiologist led to a 43% reduction in DAP and a 25% decrease in FT (*p* < 0.05, respectively). **Conclusions:** To mitigate X-radiation exposure, it is advisable for radiologists to undergo thorough training, and, ideally, the same radiologist should conduct both the TARE and the TARE-EVA. While the use of glass spheres may decrease intraarterial CA, it does not significantly impact periprocedural X-ray exposure.

## 1. Introduction

Interventional radiology is a dynamically advancing field characterized by progressively intricate and prolonged procedures. Among medical personnel, interventions guided by fluoroscopy contribute to the highest levels of occupational radiation exposure [1,2,3]. The existing literature underscores the significant risks associated with occupational X-ray exposure. A cross-sectional study conducted in France involved 106 interventional cardiologists and 99 unexposed controls. Notably, posterior subcapsular lens opacities were more prevalent among interventional cardiologists, and the risk escalated with prolonged exposure, though regular use of protective glasses seemed to mitigate this risk [4]. Numerous studies also indicate an elevated likelihood of developing brain tumors, malignant melanoma, and breast cancer [5,6]. Consequently, the emphasis on radiation protection, both for patients and medical staff, is steadily growing in importance [7,8].

Transarterial radioembolization (TARE) stands as a minimally invasive treatment for early-stage hepatocellular carcinoma (HCC) and the management of unresectable metastatic liver disease [9,10]. This technique involves the direct administration of microspheres carrying a ß-radiation-emitting isotope (typically Yttrium-90) into the hepatic arteries that supply the affected areas of the liver. The objective is to deliver concentrated doses of radiation specifically to the tumor while sparing healthy liver tissue [11].

There are two commercially available types of Yttrium-90-loaded microspheres: glass-based (TheraSpheres^®^ Boston Scientific, Marlborough, MA, USA) and resin-based (Sir-Spheres^®^ Sirtex Medical Limited, Sydney, Australia) [12,13]. These systems vary in size, the number of microspheres injected, and individual activity. Glass microspheres, with a smaller quantity but higher activity per sphere, carry a reduced risk of early vessel occlusion and subsequent non-target embolization. However, in large tumors, the distribution of spheres may be uneven [14]. On the other hand, resin spheres, injected in approximately 25 times greater numbers, provide a more uniform distribution but induce a more robust embolic effect. Consequently, a cautious and fluoroscopic-controlled injection, employing a “sandwich technique” alternating between the contrast agent (CA) and the therapeutics, is imperative to prevent reflux and non-target embolization stemming from early vessel occlusion [15,16]. This method may result in increased periprocedural X-radiation and contrast dosages.

To prepare for the actual TARE procedure, an initial preprocedural angiography (TARE-EVA) is conducted to assess the vascular anatomy, identify the target vessels, and exclude hepatomesenterial, hepatopulmonary, or hepatogastric shunts. Following this, a low dose of Technetium-99m-labeled macroaggregated albumin (99mTc-MAA) is administered into the target vessels, followed by scintigraphy to assess future radionucleotide deposition and rule out potential non-target radiation.

Our hypothesis posits that intricate anatomical conditions and a substantial need for embolization lead to prolonged fluoroscopy times (FT), heightened X-radiation doses, and increased intraarterial CA usage to ensure a safe and targeted tumor treatment.

Previous research has indicated that specific conditions related to the operator, such as the proficiency of the interventional radiologist, play a crucial role in influencing X-ray exposure during various brief procedures, such as the placement of central venous port catheters. However, there is a lack of data for prolonged and extensive interventions like TARE.

The aim of this study was to assess whether factors such as the radiologist’s experience, the choice of microspheres, and the performance of both the TARE-EVA and the actual TARE procedure by the same radiologist have a significant impact on radiation dose, FT, and CA dosage.

## 2. Materials and Methods

### 2.1. Study Design

In this retrospective study conducted at a single center, we analyzed datasets from 161 TARE and 164 TARE-EVA procedures, which were carried out on a total of 137 patients between January 2017 and March 2021. This study received approval from the Institutional Ethics Committee (Ethics Committee at the Medical Faculty of the University of Leipzig/585/21-ek), and written informed consent was obtained from all participating patients. All procedures adhered to the relevant guidelines and regulations. Furthermore, each case underwent thorough discussion at a certified interdisciplinary tumor conference. The choice between resin- or glass-based spheres for TARE was determined based on availability and the preferences of the radiologist. The datasets were scrutinized with respect to the type of microspheres used, the radiologist’s level of experience, and whether both TARE-EVA and TARE procedures were conducted by the same radiologist. For all TARE-EVA and TARE instances, we recorded the fluoroscopy time (FT) and the dose–area product (DAP) in micrograys per square meter (µG · m^2^), as provided by the fluoroscopy unit’s software (dose report, Phillips ClarityIQ V 1.2.5, Philips Healthcare, Best, The Netherlands). Additionally, the total amount of CA administered during the procedure (in milliliters) was documented.

To mitigate the possibility of confounding arising from variations in weight, we conducted an analysis of DAP, FT, and CA within two weight categories: “normal weight” (BMI < 25) and “overweight” (BMI > 25). We assessed the statistical independence of our three primary variables (experience, type of microspheres used, and whether TARE-EVA and TARE were performed by the same radiologist) in relation to the weight groups.

### 2.2. Technical Details

The DAP meter of this system is calibrated to meet national standards as outlined in DIN 6868-150:2013-06 [17]. Fluoroscopic images were captured at a rate of 7.5 frames per second, while digital acquisitions were conducted at a rate of 2 frames per second in all instances.

### 2.3. Experience of the Radiologist

The procedures were carried out by a total of 10 radiologists, each possessing varying levels of experience. The radiologists were categorized into two groups: certified interventional radiologists and trainee interventional radiologists, hereafter referred to as experienced and inexperienced, respectively. Certification was determined by either EBIR certification (European Board of Interventional Radiology) or DeGIR Step 2 (German Society of Interventional Radiology). To achieve certification, all radiologists had completed a minimum of 150 intraarterial procedures.

### 2.4. Procedures

All interventions were conducted in a specialized Angio suite utilizing a flat panel system (Azurion Clarity IQ, Philips Healthcare, Best, The Netherlands). The assessment procedures were carried out 5–10 days prior to the actual TARE procedure to evaluate the patient’s hepatic vasculature, lung shunting, and distribution of the microspheres. Both interventions were performed with the patient under local anesthesia and a standard dose of 5000 IU of Heparin intraarterially.

For both TARE and TARE-EVA procedures, a 4F or 5F sheath was introduced into the right common femoral artery (CFA). A diagnostic catheter was positioned in the common hepatic artery, and the target vessels were accessed using different microcatheters. During TARE-EVA, potential shunt vessels were identified and occluded, and a low dose of Technetium-99m was administered at the target position, followed by subsequent scintigraphy. Dyna-CT was performed in 87.6% of cases, depending on whether there was uncertainty about selectivity or if CT data were required for liver segmentation.

For the actual TARE, the microcatheter was placed at the exact same spot identified during TARE-EVA. In the case of resin spheres, the therapeutic agent was slowly and pulsatively administered together with CA under fluoroscopic control. Glass spheres were administered without fluoroscopic guidance until the entire dose was applied.

### 2.5. Statistical Analysis

All analyses were executed using SPSS Statistical Software (V25 IBM, Armonk, NY, USA). Normally distributed quantitative variables were expressed as mean values and standard deviations (SDs). The analysis included the Mann–Whitney U test and Chi-squared test for independence. A *p*-value < 0.05 was considered statistically significant.

## 3. Results

### 3.1. Patient Characteristics

The study cohort comprised 137 patients (96 male, aged 76 ± 22 years, with a mean body mass index (BMI) of 27.4 ± 7.2 kg/m^2^), and there were no significant differences observed between the resin and glass groups. Among these patients, 105 were diagnosed with primary liver tumors (79 with hepatocellular carcinoma (HCC) and 26 with intrahepatic cholangiocarcinoma (ICC)), while 32 patients were diagnosed with liver metastases. All patients with hepatocellular carcinomas were in either an intermediate or advanced stage. At the time of our study, TARE was primarily administered for advanced HCC stages as per the guidelines. Liver cirrhosis was present in 86% of the patients before undergoing TARE.

#### Differences in DAP, FT, and CA between Weight Groups

In the TARE-EVA, the DAP was notably higher in the overweight group, measuring 14,101 ± 17,909 µG · m^2^, compared to 6647 ± 5667 µG · m^2^ in the normal weight group (*p* < 0.001). However, there were no significant differences in FT and CA, with *p*-values of 0.06 and 0.484, respectively. In the TARE, both DAP and FT were significantly higher in the overweight group at 4378 ± 6707 µG · m^2^ and 11 ± 8.1 min, compared to 3164 ± 3461 µG · m^2^ and 16.2 ± 13.5 min, respectively. For both the TARE-EVA and the TARE, the Chi-squared test for independence demonstrated that the variables of experience and whether the TARE-EVA and the TARE were performed by the same interventional radiologist (IR) were independent of the two weight groups (*p* = 0.39, 0.45, and 0.91, respectively).

### 3.2. TARE-EVA

#### Impact of the Experience of the Radiologist

A total of 147 TARE-EVA procedures (90%) were conducted by experienced radiologists, while 17 were performed by inexperienced radiologists (10%). There were no noteworthy differences in the number of digital acquisitions (“DSA runs”) between experienced and inexperienced radiologists (16.9 ± 9.1 vs. 11.3 ± 5.1) (*p* = 0.202). Regarding the TARE-EVA, the mean overall FT was 19.9 ± 16.3 min, the mean overall DAP was 11,197 ± 17,801 µG · m^2^, and the mean overall dosage of administered CA was 62 ± 32.2 mL. Experienced radiologists exhibited lower DAP compared to inexperienced radiologists (11,298 ± 2180 µG · m^2^ vs. 13,883 ± 2180 µG · m^2^) (*p* < 0.05). FT was also significantly shorter for experienced radiologists, measuring 14.9 ± 1.2 min vs. 31.7 ± 6.4 min (*p* < 0.05). However, there were no significant differences in the amount of CA used (58.3 ± 3 mL vs. 64.4 ± 6.4 mL) between the two groups (*p* = 0.489) (refer to Table 1). In 8 cases, digital acquisitions were performed using oblique projections, with no significant differences in DAP. In 141 cases (87.6%), Dyna-CT was performed, with no significant variations observed between resin and glass or between experienced and inexperienced.

### 3.3. TARE

#### 3.3.1. Impact of the Experience of the Radiologist

A total of 145 TARE procedures (90%) were conducted by experienced radiologists, while 16 were performed by inexperienced radiologists (10%). For the TARE, the mean overall FT was 13.1 ± 10.8 min, the mean overall DAP was 3939 ± 5677 µG · m^2^, and the mean overall dosage of administered CA was 39 ± 21.8 mL.

When the TARE was performed by an experienced radiologist, the mean DAP was 3462 ± 548 µG · m^2^, which was significantly lower than the 6765 ± 1644 µG · m^2^ used by an inexperienced radiologist (*p* < 0.05). However, there were no significant differences in FT (11.2 ± 0.7 vs. 20.3 ± 6.6 min) (*p* = 0.117) and CA (36.9 ± 1.8 vs. 46.2 ± 6.2 mL) (*p* = 0.211) (refer to Table 1). Additionally, there were no significant differences in the number of DSA runs (*p* = 0.148).

#### 3.3.2. Resin Spheres vs. Glass Spheres

Resin spheres were utilized in 75 procedures (47%), while glass spheres were employed in 84 procedures (53%). In two cases, the type of microspheres could not be confirmed in the records. The mean overall FT was 14.3 ± 1.6 min when using resin spheres and 10.6 ± 1.1 min when using glass spheres (*p* < 0.05). The mean DAP was 3286 ± 479 µG · m^2^ when using resin spheres and 4276 ± 911 µG · m^2^ when using glass spheres, with no significant differences observed (*p* = 0.13). However, the dosage of CA administered was significantly higher when using resin spheres compared to glass spheres (43 ± 2.2 vs. 33.6 ± 2.1 mL; *p* < 0.05) (see Table 2).

#### 3.3.3. Impact of TARE-EVA and TARE Performed by the Same Radiologist

In 66 cases, the procedures were conducted by the same radiologist (41%), while in 95 cases, they were performed by different radiologists (59%). When the TARE-EVA and the TARE were carried out by different radiologists, the mean FT was 14.5 ± 1.3 min, significantly higher than when both the evaluation and the actual procedure were performed by the same radiologist, which recorded 10.9 ± 1 min (*p* < 0.05). The DAP was also significantly higher when the two procedures were conducted by different radiologists, measuring 4700 ± 734 µGy · m^2^ compared to 2693 ± 186 µGy · m^2^. However, there were no significant differences observed in the amount of CA used (41.6 ± 2.5 mL vs. 35.5 ± 1.9 mL) (*p* = 0.4) (see Table 3).

## 4. Discussion

In this study, we investigated the influence of the radiologist’s experience, the choice of microspheres, and whether the TARE-EVA and actual TARE procedures were performed by the same radiologist on the exposure to X-radiation and intraarterial CA.

Various strategies exist to mitigate occupational X-ray exposure [18,19,20]. A critical step involves avoiding direct X-radiation exposure by keeping hands outside the fluoroscopy field, thus primarily limiting occupational X-ray exposure to scatter radiation. Lead aprons with thyroid shields or lead-coated glasses play a crucial role in shielding the body from scatter radiation. However, certain body parts, particularly the arms and the head, remain unshielded and susceptible to high doses of radiation.

To quantify occupational X-ray exposure, both the ICRP and CIRSE Guidelines recommend the use of two personal dosimeters—one positioned under the lead apron and another near the eye lens. Neto et al. go further, suggesting the use of a dosimeter under and over the lead apron to ensure a more precise measurement of body dose [21]. In interventions where the radiologist’s hands are close to the fluoroscopy field, the consideration of a third ring dosimeter is advised [22,23].

Previous research has highlighted the significant role of a radiologist’s experience in reducing periprocedural radiation. For instance, Joncyk et al. demonstrated that senior radiologists required notably less FT (a reduction of 42%) and DAP (a reduction of 16%) for central venous port catheter implantation [24]. Similarly, Xu et al. found that, when radiology residents performed central venous catheter implantations, compared to staff radiologists, FT nearly doubled [25]. In the context of extensive interventions like hepatic chemosaturation, Ebel et al. revealed that, after accumulating experience with seven or more procedures, radiologists needed less FT and DAP, with a mean reduction in DAP of 29% and a mean reduction in FT of 17% [26].

In our study, we observed that board-certified radiologists required less FT/DAP in both the evaluating angiography and the actual procedure. There was a reduction of 19% in DAP and 53% in FT during the evaluation angiography and a 49% reduction in DAP in the actual TARE, with no statistically significant differences in FT. The substantial decrease in FT during the TARE-EVA supports the notion that the primary challenge in evaluation angiography is the fluoroscopy-guided identification and catheterization of target vessels, a skill heavily reliant on experience. As fluoroscopic images contribute minimally to radiation exposure compared to digital acquisitions, experience has a weaker impact on DAP.

A prospective study by Zurcher et al. limited the use of digital acquisitions in endovascular aneurysm repairs, relying mostly on fluoroscopic images, resulting in a nearly 98% reduction in periprocedural radiation dose while maintaining adequate visualization [27]. This approach could be applicable to TARE-EVA and TARE by reserving digital acquisitions for confirmatory imaging or complicated anatomical circumstances. If digital acquisitions are employed, it is advisable to minimize the number of images per second. In the actual TARE procedure, experience did not significantly influence FT, as the roadmap to the target vessels is typically established during the TARE-EVA, allowing the radiologist to rely on previously acquired images. However, DAP was significantly lower in experienced radiologists during the TARE, possibly indicating that inexperienced radiologists might use digital acquisitions more frequently for position control and take shorter intervals between each acquisition to establish new roadmaps.

There were no differences in the amount of CA dosage between the experienced and inexperienced radiologists, both in the TARE-EVA and the TARE. One of the most difficult parts of an intravascular intervention is the correct and rapid probing of the target vessels using microwires and catheters. This is where the authors perceive the most significant advantage of an experienced interventional radiologist over an inexperienced counterpart. Given that contrast medium is unnecessary during probing, thanks to the ability to generate roadmaps, the level of experience might not exert a substantial influence on CA dosage.

Joncyk et al. identified senior radiologists based on the total number of procedures, using a cutoff of 50 [24]. Conversely, Xu et al. distinguished between residents and staff radiologists [25]. In our study, we employed board certification as the benchmark for experience. EBIR certification mandates a minimum of 250 interventions, with at least 150 in the vascular domain. The DeGIR curriculum specifies a requirement of at least 100 intraarterial procedures and 75 oncologic procedures, including TARE [28,29]. This ensured that the radiologists’ experience stemmed specifically from the interventions investigated in this study.

A primary objective of our study was to assess the disparity in periprocedural radiation associated with the use of two commercially available microspheres: glass-based and resin-based. Currently, there are limited data offering a direct comparison of these two systems in terms of outcomes and safety, and as of now, no randomized studies exist. However, the existing data do suggest comparable outcomes and safety profiles [14,30]. Patients with portal vein thrombosis might benefit from the use of glass spheres, with lower toxicities and better overall survival compared to resin spheres [31]. Although a greater number of resin spheres is often employed, purportedly for a more homogeneous distribution in larger tumors, especially in patients with a higher tumor burden, there is currently no supporting data for this rationale. The requirement for meticulous fluoroscopic control during the application of resin spheres may imply a higher X-radiation dose for both the patient and the staff. Our study indeed revealed a 25% higher FT when using resin spheres; however, the periprocedural X-radiation exposure exhibited no statistically significant difference. This can be attributed to the minimal impact of fluoroscopic images on the total X-ray exposure during an intervention when compared to digital acquisitions. Additionally, there is currently no available data addressing the risk associated with an uncontrolled injection of resin spheres, highlighting the need for future studies in this regard.

Despite the lack of differences in radiation exposure, the utilization of glass spheres can result in a 15% reduction in the volume of intraarterial CA. This aspect should not be underestimated, given that complications related to CA, such as acute and chronic kidney injury, can significantly impact long-term outcomes and quality of life. A comprehensive clinical impact study conducted by Mohebri et al. involved 476 patients who experienced a contrast-associated kidney injury after Percutaneous Coronary Intervention (PCI). The study revealed a significant increase in the rate of two-year net adverse clinical events, including all-cause mortality, major bleedings, and in-stent thrombosis (HR: 1.88; 95% CI: 1.42–2) [32].

In accordance with Ebel et al.’s findings [26], we demonstrated that the execution of both the evaluation procedures and the actual TARE procedure by the same radiologist resulted in a 43% reduction in DAP and a 25% decrease in FT. Chemosaturation with hepatic perfusion, akin to TARE-EVA and TARE, also demands precise catheter positioning in order to prevent unintended systemic distribution of the chemotherapeutic agent, posing similar challenges.

Having the procedures performed by the same radiologist probably benefits two major aspects. Firstly, the radiologist is already acquainted with the patient’s individual anatomy and has devised a personalized strategy to navigate and overcome challenging anatomical conditions. Secondly, especially for digital acquisitions, which account for the majority of X-ray exposure, patient compliance (e.g., following respiratory instructions) is crucial. The authors believe that a patient who is familiar with the interventional radiologist and their communication style and is accustomed to the way instructions are articulated (e.g., holding breath) is more likely to comply. This could enhance the quality of digital acquisitions and reduce the need for repetitions.

One potential challenge in examining radiation exposure in this study was the potential for confounding due to varying body weight distributions among the groups. These differences in body weight could independently impact X-ray exposure. In coronary angiographies, a BMI exceeding 40 was found to be independently linked to a 2.1-fold increase in patient radiation dose and a 7-fold increase in physician radiation dose due to scatter radiation from other sources [33]. To ensure the credibility of our findings, we conducted an analysis of DAP, FT, and CA in two distinct weight categories: normal weight (BMI < 25) and overweight (BMI > 25). In the overweight group, both DAP and FT showed significant increases, with DAP rising by 52% in TARE-EVA and 27% in TARE, and FT increasing by 18% in TARE-EVA and 32% in TARE. Nevertheless, the Chi-squared test for independence revealed that the radiologists’ experience and whether the TARE-EVA and the TARE were performed by the same radiologist were independent and randomly distributed across the two weight groups.

The limitations of this study include its retrospective design and the relatively small size of the patient cohort. Additionally, our study was conducted at a single center, resulting in a notable imbalance between the experienced and inexperienced radiologist groups. The lack of detailed information about individual intervention circumstances, such as challenging clinical conditions or periprocedural factors, is another significant limitation. One important limitation is the absence of individual dosimetry for both patients and staff. Radiation exposure was solely assessed using the DAP meter of the angiography unit, neglecting the impact of radiation protection measures like leaden aprons and protective glasses.

It is important to note that our institution, being a university and teaching hospital, often involves procedures performed by a team consisting of one experienced and one inexperienced radiologist. Due to the retrospective nature of our study, it remains unclear how frequently the experienced radiologist intervened or took over during challenging conditions, potentially influencing factors such as FT or CA dosage. This potential bias may lead to an underestimation of the significance of experience in the outcomes.

## 5. Conclusions

In conclusion, there is a reduction in radiation dose in both TARE-EVA and TARE when carried out by experienced interventional radiologists. Although the application of resin spheres requires careful fluoroscopic control, our study revealed no significant differences in periprocedural X-radiation. However, the utilization of glass spheres can lead to a reduction in the volume of intraarterial CA. To minimize X-radiation exposure, it is advisable for the TARE and the TARE-EVA to be performed by the same radiologist.

## Figures and Tables

**Table 1:** Comparison between inexperienced and experienced IRs in TARE-EVA and TARE.

	Inexperienced IRs	Experienced IRs	*p*-Value
FT in TARE-EVA (min)	31.7 ± 6.4	14.9 ± 1.2	<0.05
FT in TARE (min)	20.3 ± 6.6	11.2 ± 0.7	0.117
DAP in TARE-EVA (µGy · m^2^)	13,883 ± 2180	11,298 ± 2180	<0.05
DAP in TARE (µGy · m^2^)	6765 ± 1644	3462 ± 548	<0.05
Contrast agent in TARE-EVA (mL)	64.4 ± 6.4	58.3 ± 3	0.489
Contrast agent in TARE (mL)	46.2 ± 6.2	36.9 ± 1.8	0.211

**Table 2:** Comparison between resin and glass microspheres.

	Resin Spheres	Glass Spheres	*p*-Value
DAP (µGy · m^2^)	3286 ± 479	4276 ± 911	0.133
FT (min)	14.3 ± 1.6	10.6 ± 1.1	<0.05
Contrast agent (mL)	43 ± 2.2	36.3 ± 2.1	<0.05

**Table 3:** Comparison of TARE-EVA and TARE when performed by the same IR or different IRs.

	Different IRs	Same IR	*p*-Value
DAP (µGy · m^2^)	4700 ± 743	2693 ± 186	<0.05
FT (min)	14.5 ± 1.3	10.9 ± 1	<0.05
Contrast agent (mL)	41.6 ± 2.5	35.5 ± 1.9	0.4

## Data Availability

The datasets used and/or analyzed during the current study are available from the corresponding author on reasonable request.

## Abbreviations

Body mass index (BMI), interventional radiologist (IR), dose–area product (DAP), fluoroscopy time (FT), contrast agent (CA), TARE evaluation (TARE-EVA), transarterial radioembolization (TARE), common femoral artery (CFA), hepatocellular carcinoma (HCC), intrahepatic cholangiocarcinoma (ICC).
